# Sodium Fluoride In Vitro Treatment Affects the Expression of Gonadotropin and Steroid Hormone Receptors in Chicken Embryonic Gonads

**DOI:** 10.3390/ani11040943

**Published:** 2021-03-26

**Authors:** Agnieszka Karolina Grzegorzewska, Ewa Grot, Andrzej Sechman

**Affiliations:** Department of Animal Physiology and Endocrinology, University of Agriculture in Krakow, Al. Mickiewicza 24/28, 30-059 Krakow, Poland; ewagrot96@wp.pl (E.G.); andrzej.sechman@urk.edu.pl (A.S.)

**Keywords:** NaF, hormone receptors, chicken embryo, gonads

## Abstract

**Simple Summary:**

Effects of in vitro sodium fluoride (NaF) treatment on the mRNA expression of luteinizing hormone receptor (*LHR*), follicle-stimulating hormone receptor (*FSHR*), estrogen receptors (*ESR1* and *ESR2*), progesterone receptor (*PGR*), and the immunolocalization of PGRs were examined in gonads of 14-day-old chicken embryos. In the ovary, the NaF treatment significantly increased mRNA levels of all investigated receptors. In the testes, the lowest applied dose of NaF (1.7 mM) significantly decreased the expression of *FSHR, ESR1*, *ESR2,* and *PGR*. Alternatively, the higher NaF dose (7.1 mM) elevated *PGR* mRNA level in the male gonad. Immunohistochemical analysis revealed that the NaF exposure increased PGR expression in the ovarian cortex, while it decreased its expression in the testes. Collectively, these data indicate that: (i) NaF may disturb the chicken embryonic development, and (ii) different mechanisms of this toxicant action exist within the female and male gonads.

**Abstract:**

Sodium fluoride (NaF), in addition to preventing dental decay may negatively affect the body. The aim of this study was to examine the effect of a 6 h in vitro treatment of gonads isolated from 14-day-old chicken embryos with NaF at doses of 1.7 (D1), 3.5 (D2), 7.1 (D3), and 14.2 mM (D4). The mRNA expression of luteinizing hormone receptor (*LHR*), follicle-stimulating hormone receptor (*FSHR*), estrogen receptors (*ESR1* and *ESR2*), progesterone receptor (*PGR*), and the immunolocalization of progesterone receptors were examined in the tissue. In the ovary, the expression of *FSHR* and *LHR* increased following the NaF treatment. In the case of *FSHR* the highest stimulatory effect was noticed in the D2 group, while the expression of *LHR* increased in a dose-dependent manner. A gradual increase in *ESR1* and *PGR* mRNA levels was also observed in the ovary following the NaF treatment, but only up to the D3 dose of NaF. The highest *ESR2* level was also found in the D3 group. In the testes, the lowest dose of NaF significantly decreased the expression of *FSHR, ESR1*, *ESR2,* and *PGR*. On the other hand, an increase in *PGR* expression was observed in the D3 group. The expression of *LHR* in the testes was not affected by the NaF treatment. Immunohistochemical analysis showed that NaF exposure increased progesterone receptor expression in the ovarian cortex, while it decreased its expression in the testes. These results reveal that NaF may disturb the chicken embryonic development and different mechanisms of this toxicant action exist within the females and males.

## 1. Introduction

Sodium fluoride (NaF) is an inorganic chemical compound, the source of which in the environment arises from the process of rock weathering and rainfall, during which large amounts of fluoride get transferred into groundwater from dust and gas pollutants of the atmosphere. Locally, the increase in the content of fluoride in the environment is caused by fertilization with phosphate fertilizers or through the presence of enamel, glass, chemical plants, and aluminum smelters [1]. 

Low-dose fluorides are beneficial to bone decaying conditions and have been used in the case of advanced osteoporosis. However, the biphasic actions of fluoride suggest that excessive systemic exposure to fluorides can lead to skeletal or dental/enamel fluorosis. NaF penetrates cell membranes. It can accumulate in organs and tissues (bones, pineal gland) and its effect depends on the dose and time of exposure [2]. Many in vitro and in vivo studies have shown a negative effect of NaF on the functioning of both the male and female reproductive systems [3,4]. NaF may adversely affect the development of the embryo and the course of pregnancy [5], it generates oxidative stress in developing fetuses [6].

The functioning of the reproductive system depends largely on the expression and location of the appropriate sex hormone binding receptors. In the chicken, gonadal sex is bipotential up to day 6 of embryogenesis (ED6). By days 8–10 of incubation, the gonads differentiate and develop as asymmetric ovaries in females (heterozygotes ZW), while in males (homozygotes ZZ) gonads develop as symmetric testes [7]. In the avian species, estrogens play an essential role in sex-dependent differentiation of the ovarian tissue and the blockage of estrogen synthesis leads to phenotypical sex-reversal in the genetic females [8,9]. The synthesis of sex steroids by embryonic gonads is regulated by pituitary gonadotropins: luteinizing hormone (LH) and follicle-stimulating hormone (FSH), while in gonadal tissue the biological action of LH and FSH is mediated by the membrane receptors LHR and FSHR. Previously, it was found that gonadotropins play an essential role in bipotential gonad differentiation as well as in the hypothalamo-pituitary-gonadal axis development in the chicken embryo [10,11]. Gonadal expression of LHR and FSHR mRNA was detected at ED4 in males and females [12]. LH stimulates estradiol synthesis and secretion from the left ovary in vivo [13] and in vitro [14]. LH in ovo injection inhibits oogonial proliferation and induces its meiotic prophase, as well as follicle formation in the ovary of a newly-hatched chicken [15]. Despite receptors binding gonadotropins, steroid hormone binding receptors play a crucial role. There are two isoforms of nuclear receptors in the gonadal tissue: Estrogen receptor α (ESR1) and β (ESR2), in which each play a different physiological role [16]. ESR2 is able to mediate the effects of estradiol in a dose dependent manner, although the level of induction was slightly lower than that obtained with ESR1 [17]. The ESR1 activity may be associated with proliferation, while ESR2 with the stimulation of apoptosis and cell differentiation [18,19]. Earlier studies showed a higher expression of ESR1 than ESR2 mRNA in the ovary of the laying hen [20,21] and in the chorioallantoic membrane of chicken embryos in ED12, ED15, and ED18 [22].

Progesterone, another steroid hormone, may initiate meiotic division of germ cells in both male and female chicken embryos [23]. Two isoforms of the progesterone receptor (PGR) have been described in chickens: The full-length form (PGR-B, 110 kDa) and the N-terminal truncated form (PGR-A, 79 kDa) [24,25]. In oviparous species, progesterone is necessary for the full development of the oviduct and for the synthesis of egg white proteins (avidin) in estrogen-stimulated oviductal function [26,27]. Yoshimura and Bahr [28] demonstrated PGRs presence before ovulation within the cells of blood vessels and endothelial cells. PGR-B dominates in the chicken ovary, playing a key role in the sexual maturation of hens, the development of the ovarian follicle, and ovulation [29]. In mammals, the PGR influences the female secondary sexual characteristics such as breast structure and the maintenance of pregnancy [30]. This receptor is present in the central nervous system, ovary, and uterus [31].

It has been found that both the PGR and ESR may play a role in breast cancer cell proliferation and their expression level is an important indicator of health status and a marker in the diagnosis of breast cancer [32]. The chicken embryo may be used as an alternative model in angiogenesis and metastasis investigations as well as in toxicological studies [33,34].

The mechanism of the influence of NaF on the developing reproductive system of birds is not fully understood, although too much fluoride in the food consumed by chickens may reduce the number and the mass of the eggs laid [35]. Moreover, our recent studies revealed that a NaF in vitro treatment of the chicken embryonic ovary and testis generated oxidative stress, which manifested as the increased expression of free radical scavenging enzymes (catalase, sodium dismutase, nuclear respiratory factor) [36]. The aim of this study was to evaluate the effect of NaF in vitro exposure on the mRNA expression of gonadotropin and steroid receptors and the immunolocalization of PGR, in the gonads of the chicken embryo.

## 2. Materials and Methods

### 2.1. Reagents Used in the Research

Eagle’s medium (Biomed, Lublin, Poland); BSA and antibiotic antimycotic solution (AAS) (Merck KGaA, Darmstadt, Germany); NaF (Sigma-Aldrich, St. Louis, MO, USA); StayRNA (A&A Biotechnology, Gdynia, Poland); TRI-Reagent (MRC Inc., Cincinnati, OH, USA), High Capacity cDNA Reverse Transcription Kit (Thermo Fisher Scientific, Waltham, MA, USA); Primers (IBB PAN, Warsaw, Poland); 5× HOT FIREPol EvaGreen qPCR Mix Plus (ROX) (Solis BioDyne, Tartu, Estonia). Primary antibody: Mouse monoclonal anti-PGR (hPRa 2; Immunogen: PGR from a human endometrial carcinoma (EnCa 101) grown in athymic mice) (MS5-12642, Thermo Fisher). Secondary antibody: Goat anti-mouse AlexaFluor 555 conjugated (A-21424, Thermo Fisher); VECTASHIELD^®^ Hardset™ Antifade Mounting Medium with DAPI (Vector Laboratories, Inc., Burlingame, CA, USA). Other reagents were purchased from Chempur (Piekary Slaskie, Poland), Warchem (Marki, Poland), and Sigma-Aldrich (St. Louis, MO, USA).

The research was carried out on fertilized eggs (*n* = 140) of Leghorn hens purchased from a local breeder (Tarnów, Poland). The eggs were incubated in an automatic incubator: Brinsea 190 Advance, under standard conditions: 37.5 °C, 55% humidity.

Gonads (left ovary and two testes) were isolated from chicken embryos on day 14 of development. The collected gonads were placed on a 24-well plate in 1 mL of Eagle’s medium supplemented with 0.05% bovine serum albumin and 2 μL of antibiotic-antimycotic solution (*n* = 6 ovaries or testes for each experimental group, separately for RNA and for immunohistochemical analysis). Tissues were incubated for 6 h at 38 °C (5% CO_2_) in the Eagle’s medium supplemented with NaF at the four following doses: 1.7 (D1), 3.5 (D2), 7.1 (D3), and 14.2 mM (D4). The concentrations of NaF in the medium were established based on previous research data [37,38].

Following incubation, the tissues were placed in StayRNA (A&A Biotechnology, Gdynia, Poland) until the RNA was completely isolated or were fixed with 10% formalin and then embedded in paraffin. To confirm the obtained results, the three independent experiments were carried out.

### 2.2. Gene Expression Analysis (RNA Isolation, Reverse Transcription Reaction, and qPCR Reaction)

RNA was isolated with the TRI-Reagent reagent according to the method of Chomczyński and Sacchi [39]. The quality and concentration of the isolated RNA were determined by spectrophotometric analysis at wavelengths 260 and 280 nm. Reverse transcription reactions were performed in accordance with the kits manufacturer’s recommendation (the reaction mixture contained: 4.2 µL of sterile water, 2 µL 10 × RT buffer, 0.8 µL 25 × dNTP MIX (100 nM), 2 µL 10 × RT primer (random primer), 1 µL MultiScribeTM reverse transcriptase, 2 µg of total RNA in 10 µL of water). Reverse transcription reactions were performed in a thermocycler (Personal Thermal cycler, Eppendorf, Germany) in the cycle: 25 °C—10 min, 37 °C—120 min, 85 °C—5 min. The obtained cDNA constituting a template for the qPCR reaction was stored at −20 °C.

qPCR reactions were performed in a 96-well thermal cycler (StepOne Plus, Applied Biosystems Foster City, CA, USA). The GAPDH gene was used as a reference gene. The following program was used: 15 min at 95 °C, 40 cycles: 15 s at 95 °C, 20 s at 62 °C, and 20 s at 72 °C for the 10 μL reaction mixture containing: 2 µL of 5 × Hot FIREPol Eva Green qPCR Mix Plus, 0.12 µL of primers (10 pmol/µL) and 1 µL of cDNA (10-fold diluted sample from RT reaction). A duplicate was performed for each sample. The relative number of genes analyzed was calculated by normalizing with the GAPDH reference gene (Primers were synthesized according to the Khillare [40] sequences. Specification, see Table 1). The expression was calculated using the 2^-ΔΔCt^ method. The StepOne program was used for the quantification.

### 2.3. Immunohistochemical Analysis of Steroid Hormone Receptors

Fragments of the examined tissues (thickness: 6 μm, sliced using a semi-automatic RM2245 rotary microtome Leica) from the ovaries and testis of domestic chicken embryos were deparaffinized in xylene, then hydrated by passing through alcohol solutions of decreasing concentrations (from 100% absolute to 75%). The slides were rinsed with water, placed in a solution containing hydrogen peroxide and methanol to inactivate the endogenous peroxidase. The preparations were then heated in a citrate buffer (pH 6.0) at 75 °C for 20 min, then rinsed with a TBS buffer. After that, goat serum was applied to the slides at room temperature for 90 min. The serum was removed and the slides were then incubated overnight at 4 °C with antibody against PGR (Mouse monoclonal anti-PGR, MS5-12642, Thermo Fisher, dilution 1:50). The negative control was performed by the replacement of the primary antibody by TBST. The tissues were then washed three times in TBS and incubated for 1.5 h with the secondary fluorescent antibody (Goat anti-mouse AlexaFluor 555 conjugated, A-21424, Thermo Fisher, dilution 1:150). In the last step, the slides were sealed with (VECTASHIELD^®^Hardset^TM^ Antifade Mounting Medium with DAPI. The preparations were observed and analyzed (*n* = 6 in each group) using an AxioScope fluorescence light microscope with an Axiocam 503 camera and ZEN 2.3 pro software (Carl Zeiss, Oberkochen, Germany). In the negative control without primary antibody against PGR, the erythrocyte autofluorescence can be seen. Therefore, in order to discriminate the positive signal of PGR and the negative one emitted by erythrocytes, additionally green autofluorescence, emitted only by erythrocytes, was analyzed (see Supplementary Materials 1 and 2).

Since the applied antibodies against androgen receptor (PA1-110, Androgen Receptor Polyclonal Antibody, Thermo Fisher Scientific, Waltham, MA, USA) and ESR1 (PA1-308, Estrogen Receptor alpha Polyclonal Antibody, Thermo Fisher Scientific, Waltham, MA, USA) were not specific and did not exert cross reactivity with chicken receptor proteins, we decided to present only the results of the immunohistochemical analysis of PGR in the ovary and testes.

### 2.4. Statistical Analysis

The results were statistically analyzed by two-way ANOVA (effect of sex treatment) followed by Duncan’s multiple range test. The values are expressed as mean ± SEM (*n* = 6) and considered significantly different at *p* < 0.05. Calculations were performed using SigmaStat (Systat Software Inc., San Jose, CA, USA).

## 3. Results

**Figure 1:**
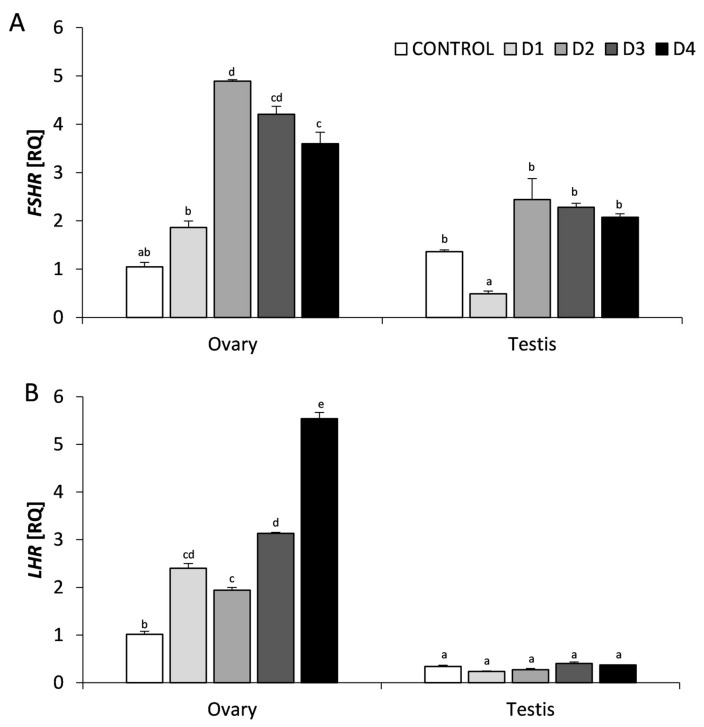
The mRNA expression of genes encoding gonadotropin receptors: follicle stimulating hormone receptor (*FSHR*) (**A**) and luteinizing hormone receptor (*LHR*) (**B**), in the ovary and testes of chicken embryos at the 14th day of embryogenesis, following an in vitro treatment with increasing doses of sodium fluoride (NaF). Each value represents the mean ± SEM from *n* = 6 (six embryonic gonads). Data of gene expression represent the mean of relative quantity (RQ), standardized to control expression in the ovary (RQ = 1). Statistical differences (*p* < 0.05) are marked by letters.

**Figure 2:**
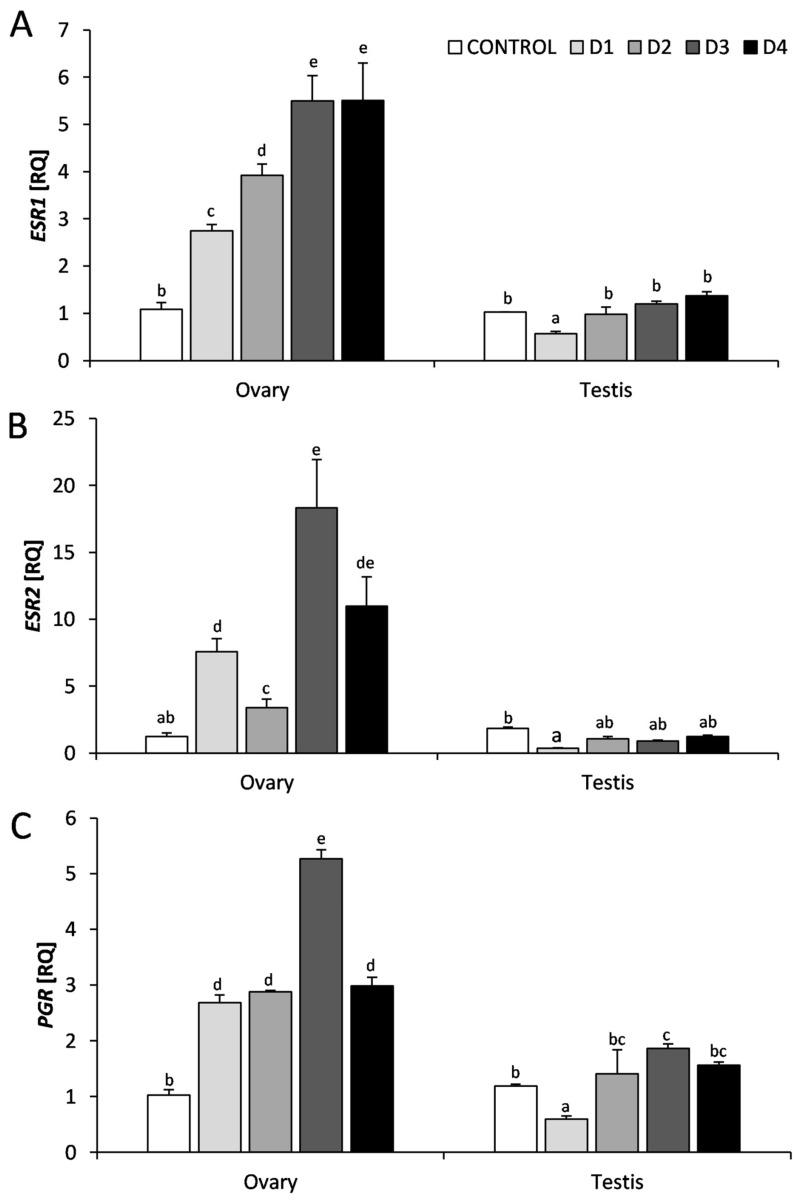
The mRNA expression of genes encoding estrogen receptors α (*ESR1*) (**A**) and β (*ESR2*) (**B**), and progesterone receptor (*PGR*) (**C**), in the ovary and testes of chicken embryos at the 14th day of embryogenesis, following an in vitro treatment with increasing doses of NaF. Each value represents the mean ± SEM from *n* = 6 (six embryonic gonads). Data of gene expression represent the mean of relative quantity (RQ), standardized to control expression in the ovary (RQ = 1). Statistical differences (*p* < 0.05) are marked by letters.

The mRNA expression of *FSHR, LHR, ESR1*, *ESR2,* and *PGR* was demonstrated in the ovary and testes of chicken embryos in all experimental groups (Figure 1 and Figure 2). In the control group, there were no significant differences in the expression of *FSHR*, *ESR1*, *ESR2,* and *PGR* genes in the female and male gonads, however with respect to the LHR gene, a lower mRNA expression was noticed in the testis. In the groups exposed to NaF, the changes in the expression of these genes were dependent on the NaF dose and the type of gonad.

In the NaF exposed ovary, the expression of *FSHR* mRNA was significantly higher in comparison with the control group, the greatest effect was noticed in the group exposed to the D2 dose of NaF (*p* < 0.01). In the testes, only in the case of the lowest dose of NaF (D1) a reduction in *FSHR* mRNA expression was observed (*p* < 0.01), the remaining NaF doses did not affect the expression of this gene (Figure 1A). The expression of *LHR* mRNA in the NaF-treated ovary gradually increased in a dose-dependent manner, reaching the highest level in the group exposed to the D4 dose (*p* < 0.01). In the testes, the NaF-treatment did not affect the mRNA expression of this gene (Figure 1B).

In the ovary, the expression of *ESR1* mRNA increased dose-dependently, reaching the highest levels in the groups exposed to the two highest doses of NaF (D3 and D4) (*p* < 0.01). In the testes, only the lowest dose of NaF (D1) reduced the *ESR1* mRNA expression (*p* < 0.05), the other doses did not affect the expression of this gene (Figure 2A). The expression of *ESR2* mRNA in the ovary was significantly (*p* < 0.05–0.01) higher in the groups exposed to NaF compared to the control group, but no dose-dependence was observed here. The D3 dose of NaF was the most effective for this receptor, followed by D4, D1, and the least effective dose of D2. In the testis, only the D1 dose of NaF (as in the case of *ESR1*) decreased the *ESR2* mRNA expression (*p* < 0.01). The higher NaF doses did not significantly affect the expression of this gene (Figure 2B).

The expression of *PGR* mRNA in the ovary in all groups exposed to NaF was significantly higher than in the control group (*p* < 0.01). The most effective was the D3 dose of NaF and the other NaF doses evoked similar stimulatory effects. In the testes, the D1 dose of NaF reduced the expression of *PGR* mRNA (*p* < 0.01), while the D3 dose increased its expression (*p* < 0.05; Figure 2C).

**Figure 3:**
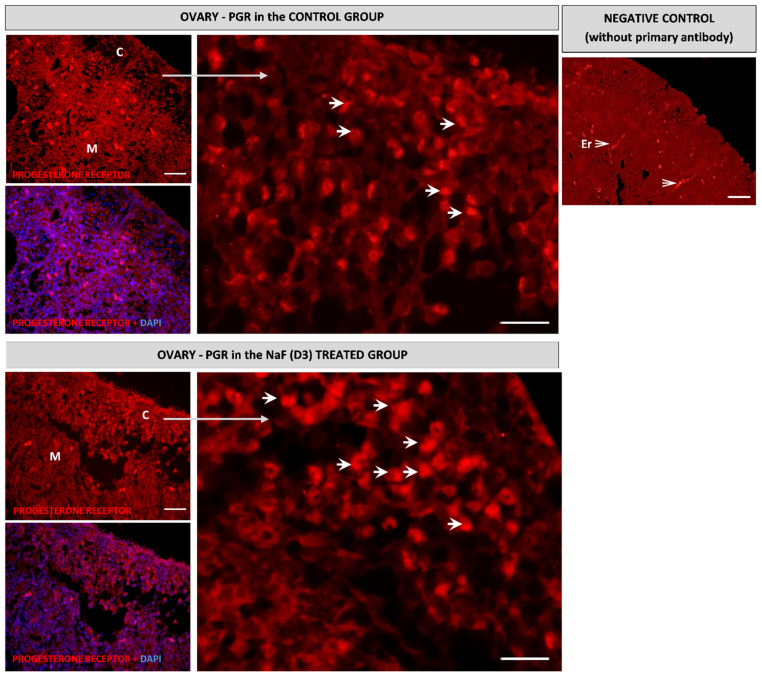
Immunolocalization of the progesterone receptor (PGR) in the control and NaF treated chicken embryonic ovaries. M: Ovarian medulla; C: Ovarian cortex; Er: Erythrocyte (autofluorescence, see Supplementary Materials 1 and 2). Arrows: Immunopositive reaction specific for the progesterone receptor (red fluorescence); DAPI: Blue fluorescence of cell nuclei. Scale bar = 100 µm.

**Figure 4:**
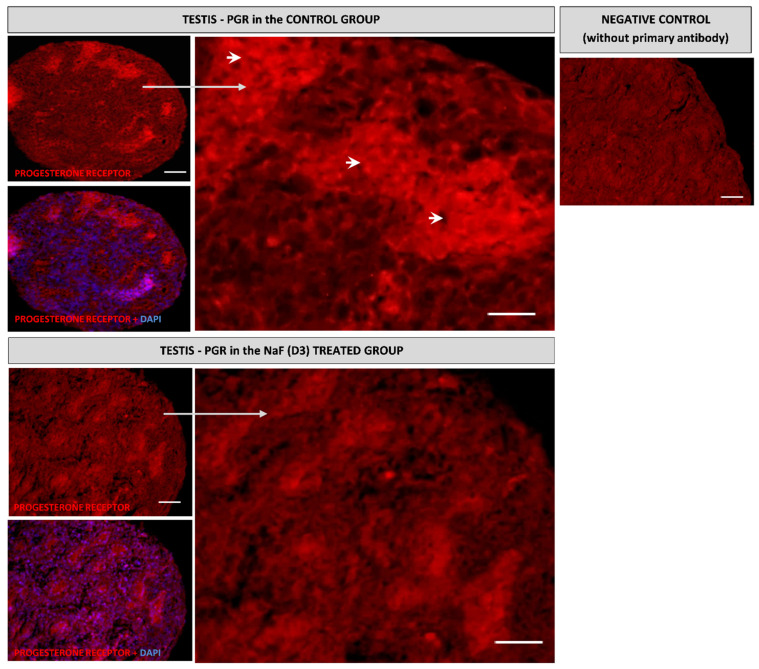
Immunolocalization of the progesterone receptor (PGR) in the control and NaF treated chicken embryonic testes exhibiting a developed medulla, characterized by seminiferous tubules (ST) with Sertoli cells and prospermatogonia. Arrows: Immunopositive reaction specific for the progesterone receptor (red fluorescence); DAPI: Blue fluorescence of cell nuclei. Scale bar = 100 µm.

The immunohistochemical analysis showed that the progesterone receptor was localized in the cortex of the embryonic ovary, while very weak immunopositive cells were found in the ovarian medulla. In the group exposed to NaF (D3), a significant increase in the expression of the progesterone receptor in the ovarian cortex was demonstrated (Figure 3). In the testes, the expression of this protein was demonstrated in developed seminiferous tubules of the medulla in the control group, while the group treated with NaF (D3) showed a significantly lower expression of this receptor (Figure 4).

## 4. Discussion

It is well known that progesterone and estradiol regulate ovulation [29,41], sexual, and breeding behavior in birds [42]. The hypothalamo-pituitary-gonadal axis development depends on specific signaling pathway activation during early embryogenesis, therefore, disorders in the expression of key receptors may disrupt the physiological functions of the organism. In the present study, the mRNA expression of LHR was significantly higher in the ovary than in the testes, while the FSHR had a similar level in both sexes. In the chicken embryo, the expression of FSHβ mRNA in the hypophysis [43] and the plasma FSH concentration [44] was higher in males than in females. In the results presented here, LHR expression was higher than FSHR expression. Similar results were obtained by Grzegorzewska et al. [43] in the chicken embryonic gonads at ED11 and ED17.

Our experiment revealed that NaF stimulates mRNA expression of gonadotropin hormone receptors (FSHR and LHR) in the chicken embryonic ovary. Previously, Zhou et al. [2,45] found that NaF at higher doses downregulates FSHR and LHR protein expression in female rats. These discrepancies may be explained by the applied NaF dose, animal model, and tissue used in the experiment.

In chicken embryonic testes, low doses of NaF decreased FSHR expression, but did not change LHR mRNA levels. Chaithra et al. [46] showed that low doses of 0.1 mg/mL of NaF can significantly affect human sperm motility, while higher doses of 10 and 100 mg/mL caused the complete loss of sperm motility and erroneous sperm formation. These results show that NaF affects male fertility and reproduction. They also indicate the high sensitivity of males to the effects of NaF. This compound, even in low doses, can lead to a decrease in fertility and problems with conception. However, too high a dose may reduce protein expression. Miranda et al. [47] showed that the functioning of mice is influenced by doses of NaF with lower values, equal to 0.01 and 0.05 mg/mL. In our studies, the dose-dependence was observed only in the female gonads, however, only up to a certain dose limit, above which the effect of fluoride on the ovary decreased.

Our experiment showed expression of mRNA of *ESR1*, *ESR2,* and *PGR* both in the ovary and testes of the chicken embryo on the 14th day of embryogenesis. The mRNA expression of *ESR1*, *ESR2,* and *PGR* was comparable in the female and male gonads. Some literature data indicate that the expression of estrogen receptors is greater in female than in male gonads already on day 10 of embryogenesis [48]. Due to the fact that sexual differentiation starts on around the 5th day of chicken embryogenesis [49], the increase in ESR1 expression in the ovary stimulates the division of cells in the granulosa layer to increase their number [50]. On the other hand, Yamamoto et al. [51] showed a similar level of *ESR1* mRNA expression in male and female chicken gonads between 5.5 and 8.5 days of embryogenesis, indicating that they are bipotential at the beginning of embryogenesis. Differences in *PGR* expression in male and female embryos appear between the 6th and 10th day of development, these receptors are located mainly in the left ovary [52]. The expression of *PGR* was found also in the oviduct of the egg laying hen [53]. It is difficult to explain the discrepancies between the obtained results and the literature data. Perhaps the lack of differences in gene expression between male and female gonads at the 14th day of embryogenesis resulted from the specificity of the experimental methodology or the analytical methods used.

In female birds, estradiol and progesterone are involved in the differentiation of both embryonic and somatic cells in the ovary [54]. In our study, both in male and female gonads, *ESR1* mRNA expression was higher than *ESR2* (RQ = 0.69 vs. 0.06 in the ovary, 0.65 vs. 0.09 in the testes). Hrabia et al. [20] showed a higher expression of the *ESR1* than *ESR2* isoform in the ovary of the laying hen, whereas the dominance of the *ESR2* isoform was detected by Oliveira et al. [55] in rooster testes. It has been found that ESR1 and ESR2 receptors also exert different functions in testes. In Japanese quails, ESR1 is responsible for the control and course of spermatogenesis [56]. In male rats, it was shown that treatment with an ESR1 agonist causes an arrest in the differentiation of round spermatids into elongated spermatids, mainly due to the downregulation of genes involved in spermiogenesis, while ESR2 agonist administration reduces sperm counts due to spermiation failure and spermatocyte apoptosis [57]. Knowledge about the distribution of ESR2 in avian tissues is currently limited. In rats, the highest *ESR2* mRNA levels were found both in the ovary and the prostate epithelium [58], as well as in several regions of the anterior hypothalamus [59]. In mice, the highest mRNA expression of this receptor was noticed in the ovary, prostate, and epididymis and in the hypothalamus and lungs in both sexes [60]. In humans, the *ESR2* transcript is abundantly expressed in the testes, ovaries, and thymus [17].

This study showed a substantial effect of NaF on the mRNA expression of steroid hormone receptors in the ovary. In comparison to the control group, a significant increase in *ESR1*, *ESR2,* and *PGR* receptor expression was observed in the embryonic ovary following exposure to all doses of NaF. On the other hand, in the testes a significant decrease in the expression of *ESR1* and *ESR2* mRNAs was demonstrated for the D1 dose of NaF, the other NaF doses had no effect on the expression of these genes. *PGR* mRNA expression was also significantly reduced in the testes exposed to the D1 dose, however the D3 dose of NaF markedly elevated this gene expression. NaF at the lowest concentration reduced the expression of *ESR2* in the embryonic testis, which according to Oliveira et al. [52] dominates in the chicken male gonads. These results may indicate the inhibitory effect of this compound on male gonads and the functioning of the male reproductive system.

Zhou et al. [45] revealed that the 12 weeks treatment of female rats with NaF (at doses of 100 and 200 mg/L of drinking water) resulted in an increase in the expression of the progesterone receptor protein, ESR1 protein, and LHR protein. These changes were accompanied by a significant decrease in the FSH receptor protein and the serum concentration of estradiol and progesterone. Another study by this group (Zhou et al. [2]) demonstrated that 6 months exposure of female rats to NaF led to a significant decrease in the concentration of reproductive hormones, with a concomitant reduction of the total number of follicles and inhibition of follicular maturation. These results suggest that fluoride affects the reproductive function not only at the hormone receptor level, but also may affect steroid hormone synthesis and secretion. It is worth emphasizing that the chicken embryo is extremely sensitive to the action of steroid hormones and any disturbances in cellular signal transduction evoked by exogenous factors may significantly affect the process of sexual differentiation [61,62].

Several lines of investigation showed expression of the progesterone receptor in the cells of the left ovary in both embryos, hatchling chicks, matured, and egg-laying hens [28,63], in preovulatory and postovulatory follicles [28], and in granular and interstitial cells in the ovarian tissues. In humans and rodents, the greatest number of immunopositive cells expressing the progesterone receptor was found within the epithelial cells of the oviduct and their expression were largely dependent on gonadotropins [64], whereas in testes, expression was immunolocalized in the epididymis and ductus deferens of immature and mature Japanese Quails [56].

In the present study, the expression of the progesterone receptor protein was confirmed by immunohistochemical staining of the 14th day chicken embryonic gonads. These receptors were localized in the cortex and medulla of the ovary and in developing seminiferous tubules in the testis. These data are consistent with previous investigations, which showed the localization of the progesterone receptor not only in the cortical part, where it is abundantly expressed, but also in the medulla [65]. On the other hand, Gasc [52] reported a greater level of this receptor within the medullary cells compared to the cortex of the left ovary of the chicken embryo. The immunohistochemical staining revealed that NaF stimulated PGR protein expression in the ovarian cortex, whereas in the testis it inhibited its expression. These results are in agreement with the results of the qPCR analysis, an increase in receptor expression in females and a decrease in males.

Disruption of the proper functioning of steroid hormone receptors, such as the ESR or PGR, may lead to the development of diseases: bone disorders (osteoporosis in humans), tumors (mainly breast cancer), heart and circulatory system diseases, and polycystic ovary syndrome (PCOS) [32,66]. It has been shown that the lack of ESR1 receptor expression in male and female rats affects their sterility. In males, ESR2 protein localization in germ cells, Leydig and Sertoli cells, epithelial cells, and spermatozoa was remarkable, which indicated the important role of ESR2 in the spermatogenesis process [67]. In the case of the female sex, it is also important to note that changes in the estrogen receptor may be related to the incidence of breast cancer and the response to administered drugs [68]. The effect of fluoride may be related to polymorphism in the gene encoding ESR1. This interaction was found in women [69] and men [70] living in contaminated areas. It has also been shown that too high a content of fluoride in the feed disturbs the functioning of the reproductive system in birds, leading to a reduction of egg laying [35].

NaF may inhibit the proper functioning of steroid receptors found in testicular cells at various times in the lives of males, and consequently affect the processes related to their fertility. NaF has also been found to affect cells within the reproductive system, such as Leydig cells and primary cells that are involved in the production of steroid hormones. NaF may interfere with the steroidogenesis process. It reduces the amount of essential proteins and inhibits the expression of genes involved in the formation of these compounds. Research conducted by Yilmaz et al. [71] showed that exposure of Leydig cells and primary cells to NaF reduces their survival and reduces the rate of proliferation. There is also a decrease in the amount of synthesized testosterone. The expression of the luteinizing hormone receptor is also decreased.

Fluorine compounds are of great importance to the developing organism, not only during embryogenesis, but also during adolescence. Research has shown that more frequent contact with fluoride accelerates puberty in girls. However, the studies on boys showed different results. Fluoride exposure slows down the maturation process in the male sex [72]. Fluoride also greatly affects the nervous system in an adverse way. It is very important to note that fluoride is able to penetrate the blood-brain barrier, accumulate within nerve cells, and disrupt neurological functions. It has now been found that long-term and overexposure to NaF may lead to a lower IQ or diseases such as autism in children [73]. In the case of birds, the fact that sodium fluoride may have an influence on the nervous system by disrupting the activity of the steroid hormones tested may be of importance. In birds, steroid hormones are responsible for the processes of learning, memory, and making sounds [74].

## 5. Conclusions

NaF has a wide influence on the expression of gonadotropin and sex hormone receptors in the chicken embryonic gonads. It may affect the development of the embryo, sex differentiation, and a number of physiological processes in the body. In females, it can overstimulate the reproductive system, which becomes much more sensitive to gonadotropins and sex steroids. In mammals, these changes may lead to cancer formation. In males, the decrease in steroid hormone and FSH receptor expression may reduce fertility. Since similar changes following NaF exposition have been observed in humans, the chicken embryonic model can be used in the analysis of NaF toxicity in humans too.

More research is needed to explain the molecular and physiological mechanisms of NaF action in the reproductive system, but it is clear that the extensive use of fluoride, especially in children, should be monitored for the safety of humans.

## Figures and Tables

**Table 1:** Positions of oligonucleotide primers in mRNA sequence, GenBank accession numbers, sequences of amplified gene primers, and PCR reaction product size.

Gene	Primer Sequence (5′–3′)	Position [bp]	GenBank Accession Number	Product Size [bp]
*FSHR*	ATGGAACCTGCCTGGATGAG CTTGTATGTAGACCTCGCTCTTAG	627–646 785–808	NM_205079	182
*LHR*	ATTGTGCTCCTCGTCCTC GTCTATGGCGTGGTTGTAG	1273–1290 1416–1434	AB009283	162
*ESR1*	TGCGAGCTCCAACCCTTTGGACA GGAGCGCCAGACTAAGCCGATCA	1037–1059 1343–1365	NM_205183	329
*ESR2*	TCCTGCTATGCTGAATTACAAC GGCTCTTAGGCTGCTCTG	399–420 548–565	AB036415.1	167
*PGR*	GGAAGGGCAGCACAACTATT GACACGCTGGACAGTTCTTC	2067–2086 2130–2149	NM_205262.1	83
*GAPDH*	GTGTGCCAACCCCCAATGTCTCT GCAGCAGCCTTCACTACCCTCT	752–774 827–848	NM_204305	97

*GAPDH*: Glyceraldehyde 3-phosphate dehydrogenase (housekeeping gene); *FSHR*: Follicle stimulating hormone receptor; *LHR*: Luteinizing hormone receptor; *ESR1*: Estrogen receptor α; *ESR2*: Estrogen receptor β; *PGR*: Progesterone receptor.

## Data Availability

All data, methods, and results of statistical analyses are reported in this paper. We welcome any specific inquiries.

## Supplementary Materials

The following are available online at https://www.mdpi.com/article/10.3390/ani11040943/s1. Supplementary Materials 1. Immunolocalization of the progesterone receptor (PGR) in the chicken embryonic ovary—control group. Red fluorescence—immunopositive reaction specific for the PGR and erythrocyte autofluorescence; Blue—fluorescence of cell nuclei (DAPI); Green—autofluorescence of erythrocytes. Supplementary Materials 2. Immunolocalization of the PGR in the chicken embryonic ovary—NaF (D3) treated group. Red fluorescence—immunopositive reaction specific for the PGR and erythrocyte autofluorescence; Blue—fluorescence of cell nuclei (DAPI); Green—autofluorescence of erythrocytes.
