# Supplementary material for: Sodium Fluoride In Vitro Treatment Affects the Expression of Gonadotropin and Steroid Hormone Receptors in Chicken Embryonic Gonads
Source: Animals (Basel). 2021 Mar 26;11(4):943. doi: 10.3390/ani11040943 (PMC8066272; doi:10.3390/ani11040943)
Supplement: Supplementary file 1 [file animals-11-00943-s001.pdf]

Supplementary materials 1: Chicken embryonic ovary – Control group

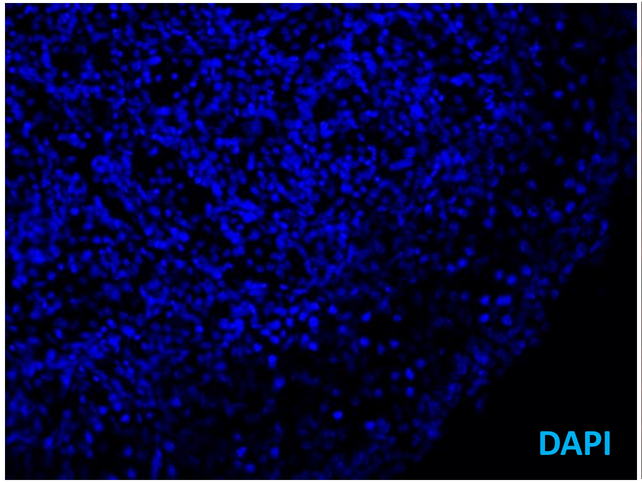

Blue fluorescence – DAPI (blue cell nuclei)

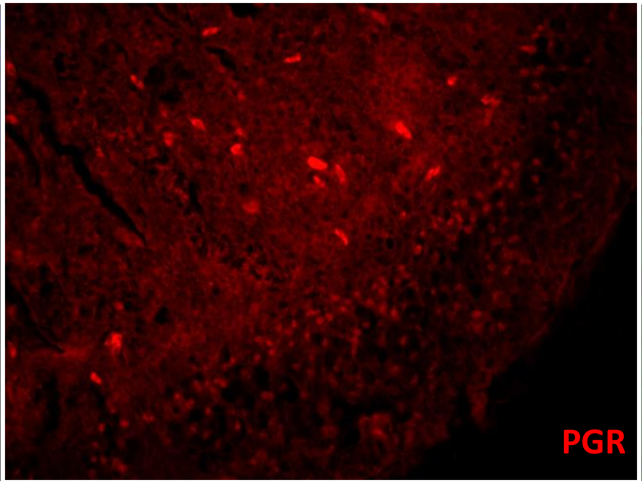

Red fluorescence – immunopositive reaction for PGR and erythrocytes autofluorescence

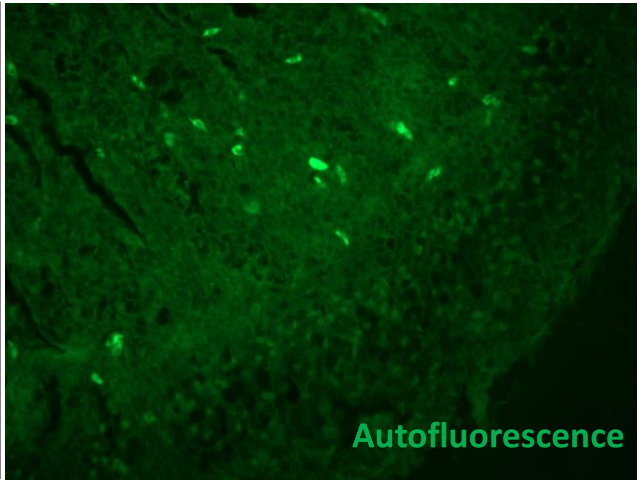

Green fluorescence – autofluorescence of erythrocytes very well visible

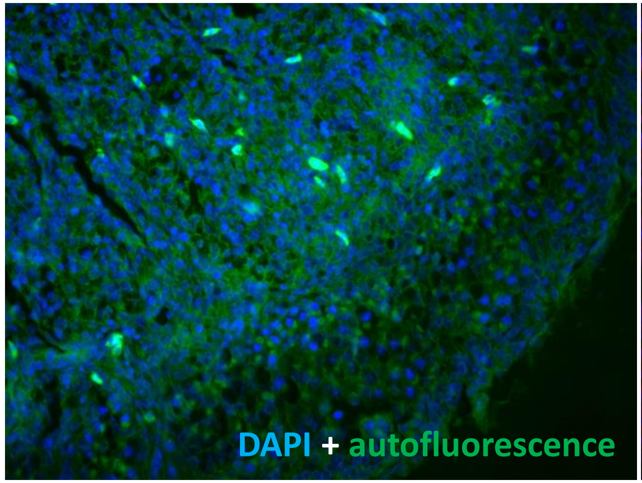

Blue + green fluorescence - nuclei and erythrocytes

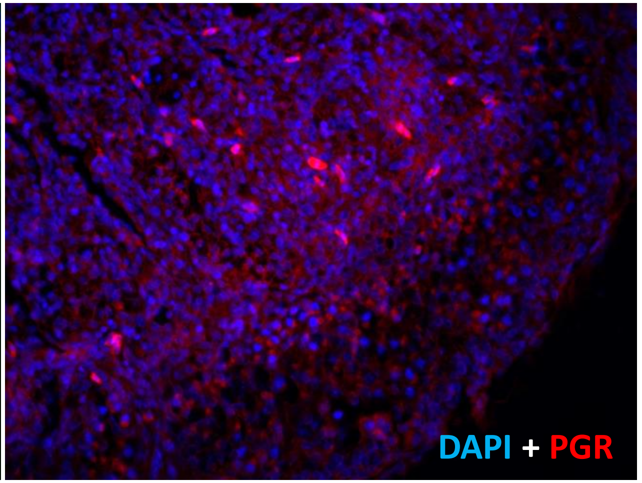

Blue + red fluorescence - nuclei, immunopositive reaction for PGR and erythrocytes

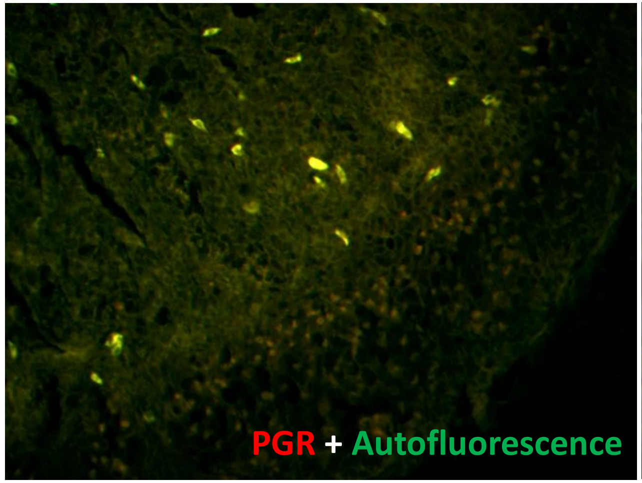

Green + red fluorescence - the same signal (colocalization). It means that **there is NO or very weak immunopositive reaction for PGR**

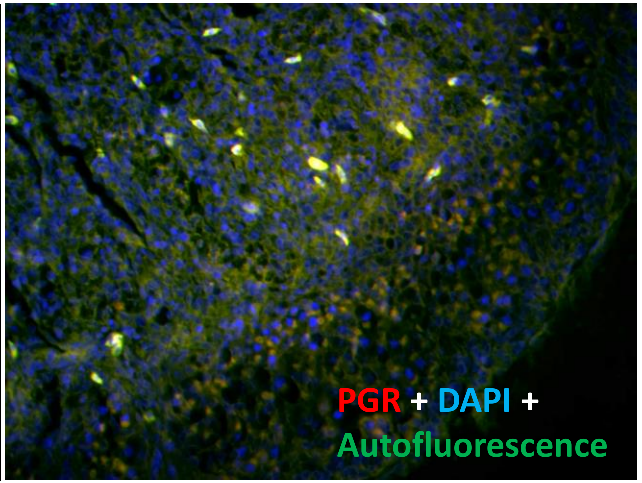

Merge (Green + red + blue fluorescence)

Supplementary materials 2: Chicken embryonic ovary – NaF (D3) treated group

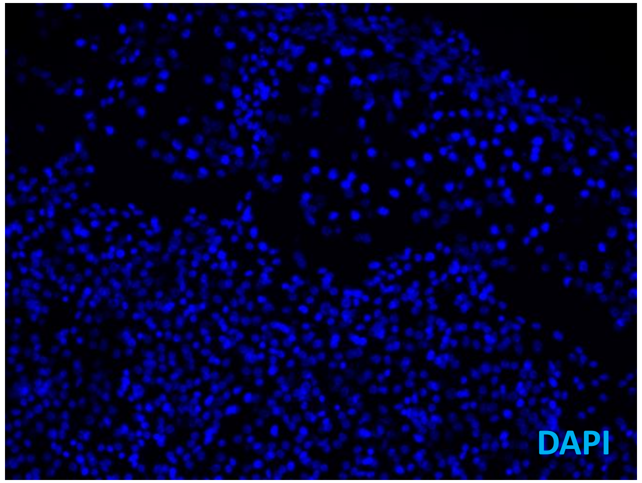

Blue fluorescence –DAPI (blue cell nuclei)

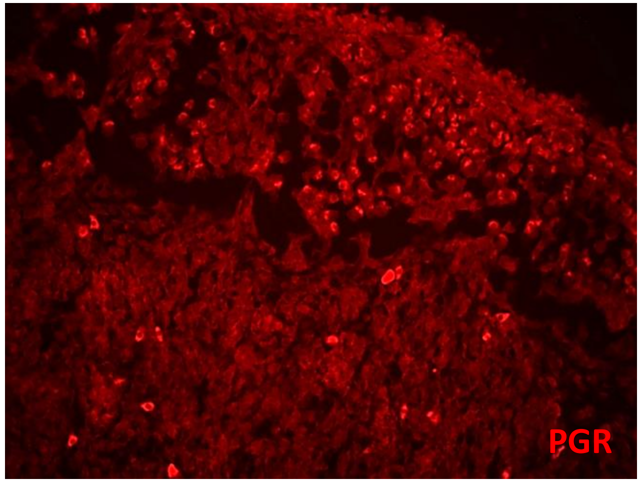

Red fluorescence – immunopositive reaction for PGR or erythrocytes autofluorescence

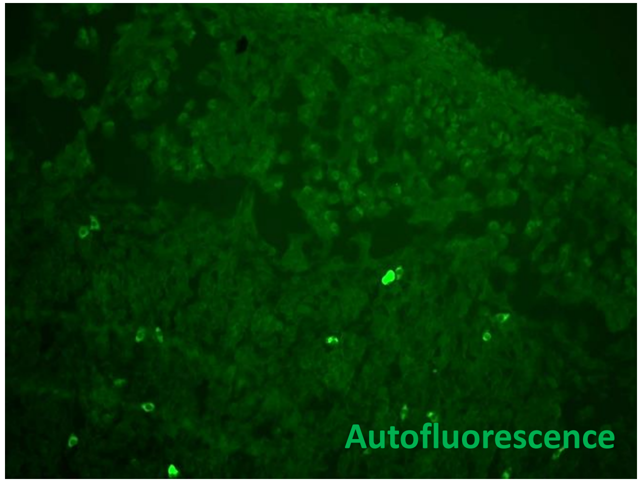

Green fluorescence – autofluorescence of erythrocytes very well visible

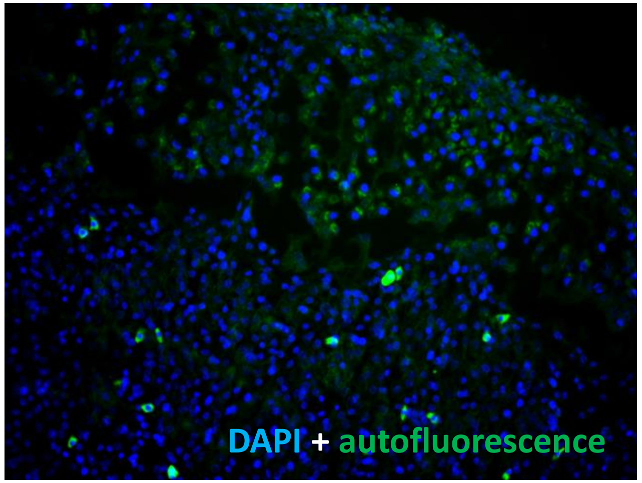

Blue + green fluorescence - nuclei and erythrocytes

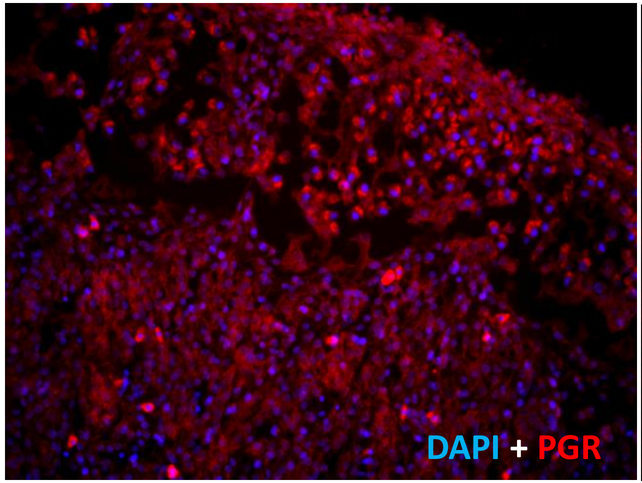

Blue + red fluorescence - nuclei, immunopositive reaction for PGR and erythrocytes

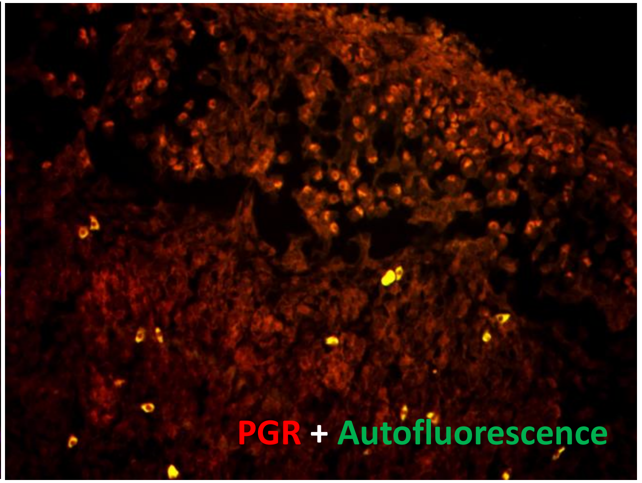

Green + red fluorescence - **NOT** the same signal (no colocalization). It means that **the reaction for PGR is specific**

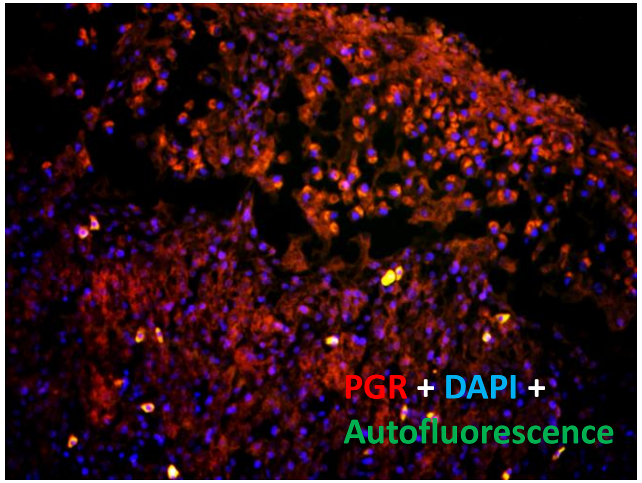

Merge (Green + red + blue fluorescence)
